# Stability-indicating Simultaneous HPTLC Method for Olanzapine and Fluoxetine in Combined Tablet Dosage Form

**DOI:** 10.4103/0250-474X.41469

**Published:** 2008

**Authors:** C. R. Shah, B. N. Suhagia, N. J. Shah, D. R. Patel, N. M. Patel

**Affiliations:** Department of Quality Assurance, Shri B. M. Shah College of Pharmaceutical Education and Research, College Campus, Modasa-383 315, India; 1 L. M. College of Pharmacy, Navrangpura, Ahmedabad-380 009, India

**Keywords:** Stability indicating method, forced degradation, olanzapine, fluoxetine, HPTLC, simultaneous estimation

## Abstract

A rapid, selective and stability-indicating high performance thin layer chromatographic method was developed and validated for the simultaneous estimation of olanzapine and fluoxetine in combined tablet dosage form. Olanzapine and fluoxetine were chromatographed on silica gel 60 F_254_ TLC plate using methanol:toluene (4:2 v/v) as the mobile phase and spectrodensitometric scanning-integration was performed at a wavelength of 233 nm using a Camag TLC Scanner III. This system was found to give compact spots for both olanzapine (R_f_ value of 0.63±0.01) and fluoxetine (R_f_ value of 0.31±0.01). The polynomial regression data for the calibration plots showed good linear relationship with r^2^=0.9995 in the concentration range of 100-800 ng/spot for olanzapine and 1000-8000 ng/spot for fluoxetine with r^2^=0.9991. The method was validated in terms of linearity, accuracy, precision, recovery and specificity. The limit of detection and the limit of quantification for the olanzapine were found to be 30 and 100 ng/spot, respectively and for fluoxetine 300 and 1000 ng/spot, respectively. Olanzapine and fluoxetine were degraded under acidic, basic and oxidation degradation conditions which showed all the peaks of degraded product were well resolved from the active pharmaceutical ingredient. Both drugs were not further degraded after thermal and photochemical degradation. The method was found to be reproducible and selective for the simultaneous estimation of olanzapine and fluoxetine. As the method could effectively separate the drugs from their degradation products, it can be employed as a stability-indicating method.

Olanzapine is an antipsychotic agent, chemically a thienobenzodiazepine described as a 2-methyl-4-(4-methyl-1-piperazinyl)-10H-thieno[2,3-b][1,5]benzodiazepine^1^ and fluoxetine is an antidepressant agent, selective serotonin reuptake inhibitor (SSRI), chemically described as a (±)-N-methyl-3-phenyl-3-[(α,α,α,-trifluoro-p-tolyl)oxy]propylamine^2^. Olanzapine in combination with fluoxetine is used in treatment-resistant depression (TRD). This combination produced a slightly smaller increase of serotonin than fluoxetine alone^3^. Literature survey reveals that few spectrophotometric, GC and HPLC methods are reported for the estimation of olanzapine and fluoxetine alone in formulations^4^ and biological samples such as urine and plasma^5^–^8^.

Forced degradation or stress studies of drug substance and products play an integral role in the development of pharmaceuticals^9^. The results of degradation studies facilitate the stability indicating method (SIM) development. Literature survey indicates that, stability indicating HPTLC method has not been developed for quantitative determination of olanzapine and fluoxetine in combined dosage form. The current ICH guidelines requires that the analysis of stability samples should be done by using stability indicating assay methods developed and validated after stress testing on drug under variety of conditions, including hydrolysis (at various pH’s), oxidation, photolysis and thermal degradation^10^. An ideal SIM is one that quantifies the drug and also resolves its degradation product. A very viable alternative for stability indicating analysis of olanzapine and fluoxetine in combined dosage form is HPTLC. The advantages of HPTLC is that several samples can be run simultaneously by using a small quantity of mobile phase unlike HPLC, thus lowering analysis time and cost per analysis^11^,^12^. In the present investigation an attempt has been made to develop accurate and precise stability indicating HPTLC method for the simultaneous estimation of olanzapine and fluoxetine in combined dosage forms^13^.

Olanzapine and fluoxetine standard were procured as a gift sample from Sun Pharmaceuticals Ltd., Baroda. Silica gel 60F_254_ TLC plates (10×10 cm, layer thickness 0.2 mm, E. Merck, Mumbai) were used as a stationary phase. All chemicals and reagents used were of analytical grade. Tablets containing 5 mg of olanzapine and 20 mg of fluoxetine (Olanex-F) were procured from a local pharmacy. A Camag HPTLC system comprising of Camag Linnomate V automatic sample applicator, Hamilton syringe (100 μl), Camag TLC Scanner 3, Camag WinCATS software, Camag Twin-trough chamber (10×10 cm) and ultrasonicator were used during study.

Olanzapine and fluoxetine (25 mg) each were weighed accurately, dissolved and diluted with methanol to obtain the final concentration of 100 μg/ml and 1000 μg/ml, respectively. Twenty tablets were weighed accurately and ground to fine powder. Weight equivalent to 25 mg of olanzapine and fluoxetine were transferred to conical flask and mixed with methanol. The solution was sonicated for 15 min. The extracts were filtered through Whatmann filter paper No. 41 and residue was washed thoroughly with methanol. The extracts and washing were pooled and transferred to a 25 ml volumetric flask and volume was made up to 25 ml with methanol. Required dilutions were made to get 100 μg/ml of olanzapine and 1000 μg/ml of fluoxetine.

TLC plates were prewashed with methanol. Activation of plates was done in an oven at 50° for 5 min. The chromatographic conditions maintained were precoated silica gel 60F_254_ aluminum sheets (10x10 cm) as stationary phase, methanol: toluene (4:2 v/v) as mobile phase, chamber and plate saturation time of 30 min, migration distance allowed was 72 mm, wavelength scanning was done at 233 nm keeping the slit dimension at 5×0.45 mm. A deuterium lamp provided the source of radiation. Three μl standard solutions of olanzapine and fluoxetine were spotted and developed. Photometric measurements were performed at 233 nm in reflectance mode with Camag TLC scanner 3 using Win CATS software.

Aliquots of 1-8 μl of standard solution of olanzapine (100 μg/ml) and 1-8 μl of fluoxetine (1000 μg/ml) were applied on the TLC plate. The TLC plate was dried, developed and analyzed photometrically as described earlier. The standard calibration curve was generated using regression analysis with Microsoft excel.

The developed method was validated in terms of linearity, accuracy, limit of detection, limit of quantification, intra-day and inter-day precision and repeatability of measurement as well as repeatability of sample application^13^. Three microlitres of sample solutions of the marketed formulation was spotted on to the same plate followed by development scanning. The analysis was repeated in triplicate. The content of the drug was calculated from the peak areas recorded.

Accurately weighed olanzapine (50 mg) and fluoxetine (500 mg) was transferred in 50 ml of volumetric flask and dissolved in methanol (25 ml). Sodium hydroxide, hydrochloric acid solution (25 ml, 1 N) and hydrogen peroxide (25 ml, 3 % v/v) were added. The final solution was transferred in 100 ml of round bottom flask and refluxed at 90±2° for 6 h. At time intervals of 0, 30, 60, 90, 120, 180 and 360 minutes, 2.5 ml of the solution was transferred in series of 25 ml of volumetric flasks and diluted to the mark with mobile phase to stop further degradation. The sample (400 ng/spot) was analyzed employing HPTLC method. For thermal stress, the drug substance in solid state was subjected to dry heat at 60° for 10 days and for photo degradation, the drug substance in solid state was exposed to UV at 254 nm for 10 days.

A solvent system that would give dense and compact spots with significant R_f_ values was desired for quantification of olanzapine and fluoxetine in pharmaceutical formulations. The mobile phase consisting of methanol: toluene (4:2 v/v) gave R_f_ values of 0.63±0.01 and 0.31±0.01 for olanzapine and fluoxetine, respectively (Table 1, (fig. 1)).

**TABLE 1:** METHOD VALIDATION PARAMETERS OF PROPOSED METHOD

Parameters	Values	
	
	Olanzapine	Fluoxetine
R_f_	0.63±0.01	0.31±0.01
Linearity range (ng/spot)	100-800	1000-8000
Correlation coefficient (r)	0.9995	0.9991
Slope (m)	10.02	5.03
Intercept (c)	8.467	189.58
Limit of detection (ng/spot)	30	300
Limit of quantification (ng/spot)	100	1000
Repeatability of application (n=7)	0.32	0.48
Repeatability of measurement (n=7)	0.19	0.09

**Fig 1 F0001:**
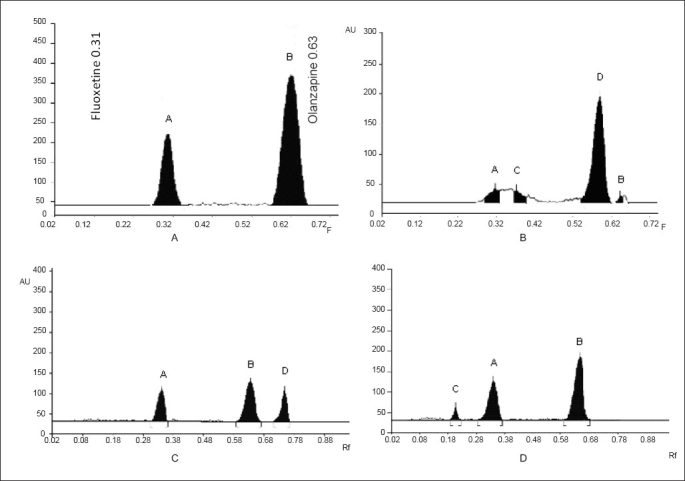
A typical HPTLC chromatograms of olanzapine and fluoxetine and their degraded products. (A) Pure drug: peak A and B are of fluoxetine and olanzapine, respectively. (B) Based induced: peak A is of fluoxetine, peak C is of fluoxetine’s degraded product, peak D is of olanzapine’s degraded product and peak B is of olanzapine. (C) Acid induced: peak A is of fluoxetine, peak B is of olanzapine and peak D is of olanzapine’s degraded product. (D) Hydrogen peroxide induced: peak C is of fluoxetine’s degraded product, peak A is of fluoxetine and peak B is of olanzapine.

The linear regression data (n=5) showed a good linear relationship over a concentration range of 100-800 ng/spot and 1000-8000 ng/spot for olanzapine and fluoxetine, respectively. The signal-to-noise ratios of 3 and 10 were considered as LOD and LOQ, respectively. The LOD and LOQ for olanzapine was found to be 30 and 100 ng/spot and for fluoxetine, 300 and 1000 ng/spot respectively. The intra-day precision was determined by analyzing standard solutions in the concentration range of 200 ng/spot to 500 ng/spot for olanzapine and 2000 ng/spot to 5000 ng/spot for fluoxetine for three times on the same day while inter-day precision was determined by analyzing corresponding standards daily for five day over a period of one week. The intra-day and inter-day coefficients of variation for both drugs were found to be in the range of 0.13-0.71 % and 0.16-0.61 %, respectively. These values indicate that the method is precise. Repeatability of sample application was assessed by spotting 3 μl of drug solution seven times on a TLC plate followed by development of plate and recording the peak area for 5 spots. The % RSD for peak area values of olanzapine and fluoxetine were found to be 0.32 and 0.48, respectively. Repeatability of measurement of peak area was determined by spotting 3 μl of olanzapine and fluoxetine solution on a TLC plate and developing the plate. The separated spot was scanned seven times without changing the position of the plate and % RSD for measurement of peak area of olanzapine and fluoxetine were found to be 0.19 and 0.09, respectively. To confirm the specificity of the proposed method, the solution of the formulation was spotted on the TLC plate, which was than developed and scanned. It was observed that the excipients present in the formulation did not interfere with the peaks of olanzapine and fluoxetine. Recovery studies of the drugs were carried out for the accuracy parameter. These studies were carried out at three levels i.e. multiple level recovery studies. Sample stock solutions from tablet formulation of 100 μg/ml for olanzapine and 1000 μg/ml for fluoxetine were prepared. Dilutions were made and recovery studies were performed. Percentage Recovery value of olanzapine and fluoxetine were found to be 99.42-100.42±0.75 % and 101.33-101.37±0.65 %, respectively while assay value of olanzapine and fluoxetine were found to be 101.5 3±1.06 % and 101.45±0.35 %, respectively. The low RSD value indicated the suitability of the method for routine analysis of olanzapine and fluoxetine in pharmaceutical dosage forms.

Degradation studies (for 0-360 min) indicate that olanzapine and fluoxetine showed good degradation in acidic and basic condition, which started to degrade after 30 min and continued up to 180 min. Fluoxetine also showed good degradation in oxidation condition, started to degrade after 30 min and continued to degrade up to 360 min, while olanzapine did not show any degradation up to 360 min. Olanzapine and fluoxetine did not show any degradation in UV light at 254 nm as well as in thermal stress at 60° up to 10 days (Table 2, ([Fig F0001]). The developed stability indicating HPTLC technique for the simultaneous estimation is simple, precise, specific, accurate and the statistical analysis proved that method is reproducible and selective for the analysis of olanzapine and fluoxetine in bulk drug and tablet formulations.

**TABLE 2:** FORCE DEGRADATION STUDY OF OLANZAPINE AND FLUOXETINE

Agent	Exposure time	Condition	% of drug remaining after degradation	
			
			Olanzapine	Fluoxetine
None	Nil	Normal	101.23	101.16
0.5 N NaOH	0-360 min	Heat	4.97	20.55
0.5 N HCl	0-360 min	Heat	39.20	48.35
3% H_2_O_2_	0-360 min	Heat	98.47	77.01
Thermal stress, 60°	10 days	Controlled oven	99.98	99.85
UV light	10 days	254 nm	99.92	99.96
